# Enabling ad-hoc reuse of private data repositories through schema extraction

**DOI:** 10.1186/s13326-020-00223-z

**Published:** 2020-07-08

**Authors:** Lars Christoph Gleim, Md Rezaul Karim, Lukas Zimmermann, Oliver Kohlbacher, Holger Stenzhorn, Stefan Decker, Oya Beyan

**Affiliations:** 1 Informatik 5, RWTH Aachen University, Ahornstr. 55, Aachen, 52062 Germany; 2 Fraunhofer FIT, Schloss Birlinghoven, Sankt Augustin, 53754 Germany; 3 Institute for Translational Bioinformatics, University Hospital Tübingen, Sand 14, Tübingen, 72076 Germany; 4 Applied Bioinformatics, Department of Computer Science, University of Tübingen, Sand 14, Tübingen, 72076 Germany; 5 Institute for Bioinformatics and Medical Informatics, University of Tübingen, Sand 14, Tübingen, 72076 Germany; 6 Quantitative Biology Center, University of Tübingen, Auf der Morgenstelle 10, Tübingen, 72076 Germany; 7 Biomolecular Interactions, Max Planck Institute for Developmental Biology, Max-Planck-Ring 5, Tübingen, 72076 Germany; 8 Institute for Medical Biometry, Epidemiology und Medical Informatics, Saarland University Medical Center, Kirrberger Str., Building 86, Homburg, 66421 Germany

**Keywords:** Semantic web, Linked data, RDF, SPARQL, Schema extraction, Privacy, Data access, Distributed systems, Query design, Personal health train, FAIR data

## Abstract

**Background:**

Sharing sensitive data across organizational boundaries is often significantly limited by legal and ethical restrictions. Regulations such as the EU General Data Protection Rules (GDPR) impose strict requirements concerning the protection of personal and privacy sensitive data. Therefore new approaches, such as the Personal Health Train initiative, are emerging to utilize data right in their original repositories, circumventing the need to transfer data.

**Results:**

Circumventing limitations of previous systems, this paper proposes a configurable and automated schema extraction and publishing approach, which enables ad-hoc SPARQL query formulation against RDF triple stores without requiring direct access to the private data. The approach is compatible with existing Semantic Web-based technologies and allows for the subsequent execution of such queries in a safe setting under the data provider’s control. Evaluation with four distinct datasets shows that a configurable amount of concise and task-relevant schema, closely describing the structure of the underlying data, was derived, enabling the schema introspection-assisted authoring of SPARQL queries.

**Conclusions:**

Automatically extracting and publishing data schema can enable the introspection-assisted creation of data selection and integration queries. In conjunction with the presented system architecture, this approach can enable reuse of data from private repositories and in settings where agreeing upon a shared schema and encoding a priori is infeasible. As such, it could provide an important step towards reuse of data from previously inaccessible sources and thus towards the proliferation of data-driven methods in the biomedical domain.

## Background

Data-driven methods play an increasingly important role for cost-efficient and timely research results and effective decision support [2] throughout numerous domain such as economics [3], education [4], manufacturing [5], healthcare and life sciences [6–8].

At the same time, the data that build the foundation of these models oftentimes underlies strict sharing requirements. For example, in the sensitive healthcare domain, although first responders, hospitals, and many other stakeholders already collect valuable data for data-driven research and treatment today, large portions of this data remain inaccessible to the majority of stakeholders – largely due to ethical, administrative, legal and political hurdles that render data sharing infeasible [9]. In practice, this leads to an inability to access large amounts of data crucial for a variety of tasks such as the optimization of decision support systems, first response systems and data-driven research. At the core of this issue lies the lack of an effective mechanism to allow for data access in a legally certain, sustainable and cost-efficient manner without extensive delays.

For example, learning health systems, allowing for data-driven research on sensitive data such as electronic health records (EHRs), have long been said to bear the potential to “fill major knowledge gaps about health care costs, the benefits and risks of drugs and procedures, geographic variations, environmental health influences, the health of special populations, and personalized medicine.” [10]. While a variety of such systems have been proposed [10–13], practical implementation has so far not become a reality, likely due to the aforementioned hurdles.

In order to enable data economy in privacy-sensitive domains and effective reuse of existing data and research, novel approaches are emerging to overcome these limitations. One of those approaches is the Personal Health Train (PHT) framework [14], which aims to bring algorithms and statistical models to data sources, rather than sharing data with the third parties such as researchers. The main benefit of this approach is its ability of utilizing all the data, including the sensitive and private information, without data having to leave the original data source. A key challenge of this approach is that data users (such as researchers) are required to develop their models without having a grasp of the actual data. Unless there are universally agreed information models and data set descriptions, there is a need to create and communicate a schema – that is information about the structure of the data – to enable writing queries for heterogeneous data resources.

This work is embedded in our ongoing efforts supporting data reuse in healthcare environments and conducted as part of the SMITH [15] and DIFUTURE [16] projects.

The key contributions of this paper consist of an automated approach for extracting task-relevant schema from RDF data sources for the efficient formulation of data selection and integration queries without direct access to the data and a corresponding integration with an information system architecture that allows for the subsequent evaluation of that query in a secure enclave.

In the following, we describes some related work and the basic foundations of our approach. Subsequently, we outline the motivation of our research, as well as the key challenges of schema extraction from sensitive data without sacrificing privacy, followed by the description of our proposed schema extraction approach from existing data in the methods section. We then present a number of evaluation results of the proposed data selection and integration methodology, based on the schema extracted from a sample use case. After a discussion of our results, we finish with a conclusion of our results and a short outlook of directions for future work.

## Related work

In order to facilitate knowledge discovery for both humans and machines, the FAIR data principles [17] have been proposed: A set of guiding principles to make research and scientific data Findable, Accessible, Interoperable, and Re-usable. These guidance principles promise to help in the discovery, access, integration and analysis of task-appropriate scientific data and associated algorithms and workflows. Thus, FAIR is gaining a lot of attention and increasing adoption.

Core to realizing these principles are Semantic Web Technologies [18], which provide a framework for data sharing and reuse by making the semantics of data machine interpretable. Particularly the directed, graph-based data model RDF [19–21] (built entirely upon the notion of statements, i.e. data in the form of subject predicate object triples) in conjunction with formal conceptualizations of information models, semantics and encoding conventions in RDF vocabularies and ontologies takes an important role.

As such, RDF Schema (RDFS) [22] and the Web Ontology Language (OWL) [23] provide a proven framework in order to describe (but not necessarily enforce) the structure and semantics of data. Substantially, RDFS introduces the concepts of classes and properties as well as basic relations between them. OWL – a computational logic-based language – extends upon these concepts in order to represent rich and complex knowledge about things, groups of things, and relations between them.

In the context of this work, we use the term ‘schema’ to refer to the semantic and structural annotation of data using especially these two vocabularies.

On the other hand, the classical notion of schema as the formal definition of the shape that data needs to comply with in order to be valid (i.e. schema validation and enforcement) also exists in the Semantic Web with the Shape Expression Language (ShEx) [24] and the Shapes Constraint Language (SHACL) [25]. At this time, there are however no established ways of sharing data shapes through public repositories and as such, in practice, they are only adapted in isolated deployments.

Nevertheless, using RDFS and OWL, it is possible to create domain-specific, optionally interoperable vocabularies and ontologies, which may declare e.g. term or concept equivalences and dependencies between each other and subsequently enable interoperability across individual encodings.

**Fig. 1:**
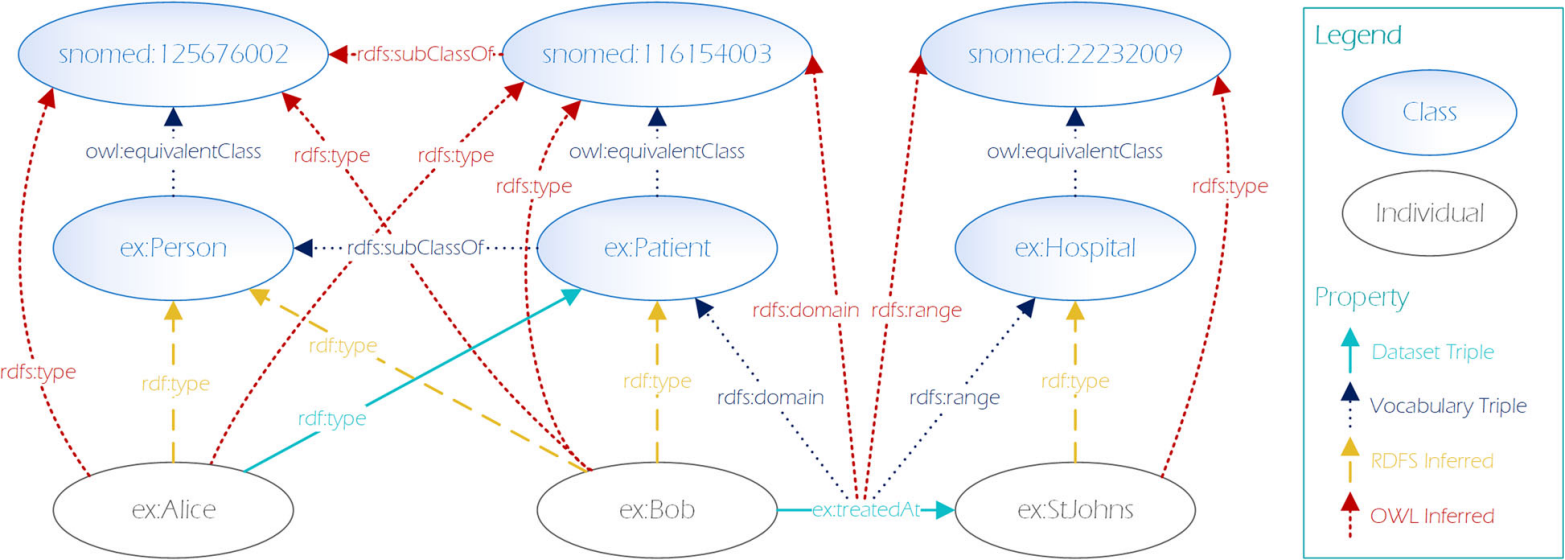
Illustration of several effects of entailment support of a SPARQL endpoint

Key to realizing the semantics described in RDFS and OWL vocabularies is the inference or entailment of implicit knowledge (inferred triples) that follow from explicit knowledge (dataset triples) via the semantics described in the corresponding vocabularies. Figure 1 illustrates some of the inferred triples that follow from the formal RDFS and OWL entailment semantics [26]. Here we assume the namespaces ex and snomed to be defined^1^.

In this example, only a small number of triples is contained in the actual dataset, while the majority of knowledge is inferred using RDFS and OWL semantics. Notably each resource is either a class, a property or an individual, i.e. either schema or data.

Popular examples of RDFS and OWL vocabularies include the Ontology for Biomedical Investigation (OBI) [27] in the biology and healthcare domain, the GoodRelations ontology [28] in eBusiness and the DCAT vocabulary [29], which is used for the general purpose metadata annotation of datasets and data catalogs.

In the context of eHealth systems, support for the Semantic Web is becoming more and more prominent with candidates such as the multilingual thesaurus SNOMEDCT [30], ongoing research efforts into an RDF specification of HL7 FHIR [31], as well as the establishment of clear guidelines for dataset descriptions such as the HCLS Community Profile [32].

Various high-quality catalogs of freely reusable vocabularies exist, allowing for the easy discovery of suitable terminology to semantically annotate data. Examples include the Linked Open Vocabulary (LOV) [33–35] and the BioPortal [36–38] project.

The related idea of using schema export and import for federated data access date back to as early as 1985 [39] but it is only recently that the idea has received more attention in the context of the Semantic Web.

Kellou-Menouer et al. [40] propose a schema discovery approach based on hierarchical clustering instead of data annotations thus leading to an approximate schema. Florenzano et al. [41], Lohmann et al. [42, 43] and Dudáš et al. [44] introduce approaches focused on schema extraction for visualization of the data structure but do not consider publishing or reuse of the extracted schema. Benedetti et al. [45, 46] propose an interesting related approach for schema extraction, visualization and query generation but do not consider interoperability issues and rely on custom mechanisms for schema storage.

### Motivation

Recently, Jochems et al. [47] and Deist et al. [48] introduced two related promising Semantic Web-based approaches in the context of the PHT initiative, founded on the key concept of bringing research to the data rather than bringing data to the research. As such the underlying information system architecture enables learning from privacy sensitive data without the data ever crossing organizational boundaries, maintaining control over the data, preserving data privacy and thereby overcoming legal and ethical issues common to other forms of data exchanges.

The general approach of this underlying system can be outlined as follows:

1. Initially, both the client and data provider agree upon a set of attributes or features, such that all participating data providers have corresponding sources of (privacy sensitive) data.
2. Then each data provider encodes their data using an (also agreed upon) ontology or vocabulary, converting it into RDF representation. This process yields proper Linked Data [49] and thus enables semantic interoperability [50].
3. The resulting RDF data is deployed to a private triple store at each location, providing a private SPARQL [51] query endpoint, which is not directly accessible by the client.
4. A SPARQL data query is then formulated based on the previously agreed upon encoding and a corresponding distributable processing algorithm defined.
5. The shared query is then executed locally at each data provider against their respective triple stores and the returned data processed using the corresponding algorithm.
6. The local results are then combined into a global one.
7. Depending on the approach, steps 5 and 6 may be further iterated.

While these approaches – introduced in the context of the PHT initiative – work well when multiple parties agree on jointly collecting, encoding and evaluating data in advance – such as is the case for conducting individual coordinated studies – they solve the issue of interoperability by agreeing on a single shared knowledge representation and encoding methodology a priori (steps 1-3 in the above process). In an optimal setting where agreeing on a single shared and global information model and encoding, reuse of diverse and existing data could always be directly accomplished with this approach.

However, to our knowledge, so far all corresponding efforts have been unsuccessful. At the time of writing the popular https://fairsharing.org/ portal indexes 1084 databases using 1183 standards, suggesting that in practice, each collected dataset and domain much rather tends to introduce its own encoding methodology.

Additionally, RDF datasets de facto often combine terms from multiple vocabularies and ontologies, sometimes deviating from the originally intended information models and encodings.

Thus when trying to reuse diverse existing data, a proper understanding of the real structure of the available data – i.e. the schema of the data – is indispensable. For a client without direct access to the data, this information is however typically not available, since its acquisition inherently relies upon inspection of the structure of the data.

Approaches, such as the PHT, depend upon ad-hoc data selection and integration facilities (step 4 of the PHT approach, corresponding to the first two steps of the classical Knowledge Discovery in Databases (KDD) process [52]) for the efficient and effective extraction of knowledge from private data sources. In order to enable the usage of such an approach with diverse existing data, suitable methods for the extraction and distribution task-specific schema, tailored specifically for the purpose of enabling ad-hoc data selection and integration, are needed.

## Methods

In this section, we propose an automated approach for extracting task-specific schema from RDF data sources in order to enable the efficient formulation of SPARQL data selection and integration queries without direct access to the data. First, we describe basic requirements for the extracted schema, as well as the fundamental idea of the schema extraction technique before subsequently introducing a number of extensions, in order to support for more generally applicable schema extraction methodology. We discuss the trade-offs to be made between different versions of the schema extraction approach and finally show how the extracted schema can be used further for the data selection and integration.

In the context of RDF data, the fundamental knowledge required for the creation of SPARQL queries for data selection and integration consists of the various rdf:type objects, the rdf:Property predicates and the structural relations between them. This information can itself be represented using Semantic Web Standards, such as RDFS, OWL, ShEx or SHACL.

While shape languages such as ShEx and SHACL are natural candidates for representing prescriptive data schema, they are designed specifically for the validation of clearly structured individual data shapes and to communicate explicit graph patterns. As such they are however not equally well suited for the formalization of the flexible schema of entire semi-structured datasets.

RDFS on the other hand provides a simple and descriptive structural annotation of the relationships between properties and classes and as such serves as a promising candidate for the task at hand.

While OWL further extends RDFS with a powerful set of description logic-based modeling primitives, the corresponding semantic complexity adds significant overhead to the schema extraction process. Especially since the extracted schema is only meant to be used for query authoring and explicitly not for reasoning, in the context of this work we generally restrict our effort to extracting schema using RDFS and the OWL owl:equivalentClass, owl:equivalentProperty and owl:sameAs predicates, which we deem most relevant in order to enable interoperability and the effective formulation of selection and integration queries.

Especially in order to ensure interoperability with existing Semantic Web technologies and compatibility with standard Semantic Web tools, such as schema-introspection-assisted SPARQL query builders, the extracted schema should thus be available as a simple RDFS and OWL vocabulary via a SPARQL endpoint.

Schema-introspection refers to the process of examining the schema definition to determine which types of entities exist, which properties are defined upon them and subsequently, what can be queried for. Since the schema needed to create data queries (e.g. using SPARQL) only contains basic structural information about the original data, it also conveys far less privacy critical information than exposing the actual data. As such it can be published publicly without privacy concerns in many scenarios.

In the following, we describe an automated approach for schema extraction from RDF data which allows for the formulation of data selection and integration queries without direct access to the data and the subsequent evaluation of that query in a secure enclave.

### Schema extraction

We propose an approach for schema extraction based on exploiting key characteristics of RDF, RDFS, and OWL. RDF data encoded in compliance with corresponding vocabularies inherently include metadata about their semantics and structural relationships.

For the schema extraction, the rdf:type relation plays the key role, as it declares data points to be instances of specific data types or, according to RDFS terminology and semantics [53], classes. Anything that is a type in the sense of occurring as the target of this relation should thus automatically becomes part of the schema as an entity of type rdfs:Class. Additionally, any property relation (that is any identifier occurring in the predicate position of a subject-predicate-object triple) which occurs in the data should be included as an entity of type rdf:Property. Finally all directly describing properties of these classes and properties should be included as well. For the scope of this work, we assume that all data in the private data repository is sensitive and should remain private.

**Entailment supported schema extraction** Assuming perfect conditions, namely proper inclusion of all used vocabularies into the triple store, correct usage of those vocabularies, as well as OWL entailment [26] support of the SPARQL endpoint providing access to the data, the entire schema of a given RDF data set can be extracted using a single simple SPARQL CONSTRUCT query as depicted in Listing 1.


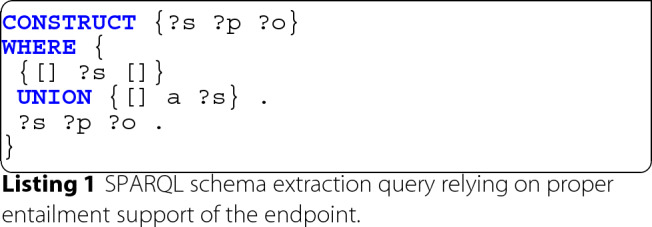


Note that we explicitly define the relevant subset of all available schema information to be that which is actually used in the data, i.e. the instantiated schema, and thus only extract that.

The preceding query constructs an RDF graph (line 1) containing all the directly describing triples ?s ?p ?o that occur in the tripe store but having only the following subjects:

1. Instantiated RDF properties ?s (line 3) which according to RDF 1.1 Semantics [53] are any IRI used in predicate position (c.f. rdfD2).
2. Instantiated RDFS classes ?s (line 4) via their occurrence as the object of a triple with rdf:type as the predicate. The fact that these are RDFS classes follows directly from the RDFS axiomatic triple rdf:type rdfs:range rdfs:Class. in conjunction with RDFS entailment pattern rdfs3 [53].

According to the SPARQL entailment regime, all the subclass relationships, transitive properties, equivalences etc. used in the data are automatically materialized (i.e. included in the dataset as inferred knowledge as illustrated in Fig. 1) and thus resolved and included too (c.f. [53, 54]).

It should be noted that the query only extracts direct properties (i.e. triples ?s ?p ?o directly related to the subject ?s) and as such, some complex constraints such as OWL disjointness axioms are not included in the extracted schema. However, as stated before, for the task of query formulation we consider this to be sufficient.

**Directly instantiated schema** Since in practice few SPARQL endpoints actually support any kind of entailment and usually do not materialize implicit triples, the applicability of this basic approach is limited. While the original query can theoretically also be executed without entailment support, it does not guarantee that all used properties and classes are annotated accordingly as rdf:Property and rdf:Class and completely ignores any resource ?s that lacks further describing triples ?s ?p ?o.

Thus, in the following we introduce several revisions of the initial extraction query 1 that allow us to reintroduce the missing triples without relying upon entailment support. Additionally, many datasets de facto employ terms from a number of different vocabularies and ontologies and deviate from the originally intended information model. Since the availability of information about domain and range of the different properties employed in the dataset is especially relevant in order to assist the query creation process, we further explicitly construct rdfs:domain and rdfs:range statements according to the property’s respective usage in the dataset.

In scenarios where it is sufficient to consider only those types and properties that are directly used in the dataset or where no information whatsoever about the employed vocabularies is available, it can be reasonable to disregard the inference generalizations and equivalences entirely. Listing 2 proposes a SPARQL query for the extraction of a corresponding schema, which closely reflects the structure of the underlying data and works even if the definitions of the employed ontologies are unavailable.

For this and all further queries, we assume standard SPARQL namespace and prefix definitions as specified by the World Wide Web Consortium’s OWL and SPARQL specifications [53, 55].


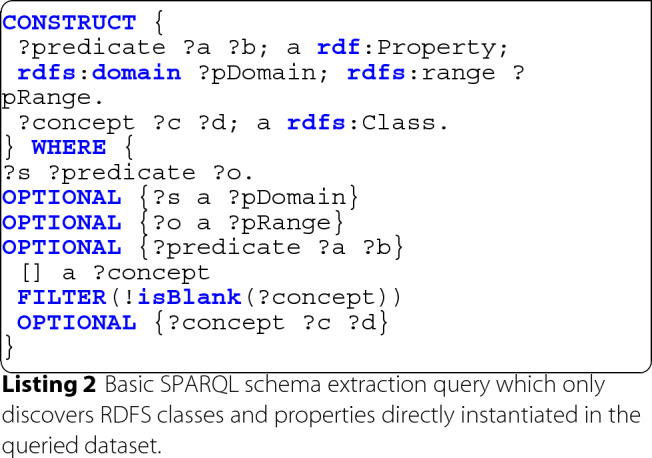


Analogously to query 1, we detect predicates as any Internationalized Resource Identifier (IRI) used in predicate position (line 5) and classes as IRIs used as objects of RDF type triples (line 9). We also include any additional information directly relating to those subjects that might be available in the dataset (lines 8 and 11). To explicitly construct rdfs:domain and rdfs:range information of the predicates, we further determine the rdf:type of each subject (line 6) and object (line 7), if available. Additionally we filter out any class declarations without an own identifier (line 10) to avoid potential referencing issues with the extracted schema. Lastly we construct the schema graph as all discovered predicates (explicitly typed as rdf:Property) and their related information (line 2) and all discovered classes (explicitly typed as rdfs:Class) and their related information (line 3).

**Fig. 2:**
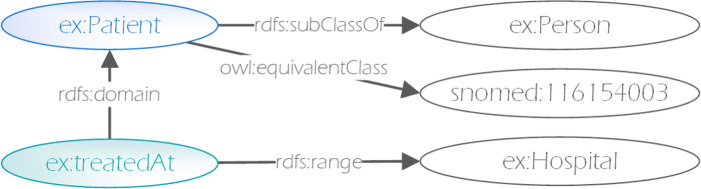
Directly instantiated schema extracted from Example 1

When applying this extraction approach to the dataset depicted in Fig. 1, we end up with the schema depicted in Fig. 2 where classes are highlighted in blue and properties in green (i.e. with implicit rdf:type triples).

Subsequently, in this exemplary use case, following the extracted schema closely one could query for instances of the ex:Patient class and their corresponding property ex:treatedAt, which however perfectly reflects the available dataset without inferred knowledge.

It should be noted, that this extracted schema is explicitly not suited for triple entailment according to RDFS semantics, due to the conjunctive nature of multiple rdfs:domain and rdfs:range definitions on properties (c.f. RDFS entailment patterns rdfs2 and rdfs3 [53]). A semantically correct alternative would be the usage of Schema.org’s schema:domainIncludes and schema:rangeIncludes properties in line 2, instead of their RDFS equivalents. However, since RDFS domain and range semantics are implemented in a variety of tools for schema exploration, visualization and assisted query authoring [56–58], while schema.org semantics are not equally well supported, we deliberately defer semantic correctness to a closer representation of the underlying data’s structure.

**Locally inferred schema** In order to re-include previously inferred information such as additional types and classes due to sub-property, subclass, domain, range or equivalence relationships, we can extract the relevant schema directly from the data and the full definitions of the employed ontologies using the SPARQL 1.1 Property Paths [59] feature, independent of entailment support or statement materialization on the endpoint.

A corresponding SPARQL query is depicted in Listing 3.


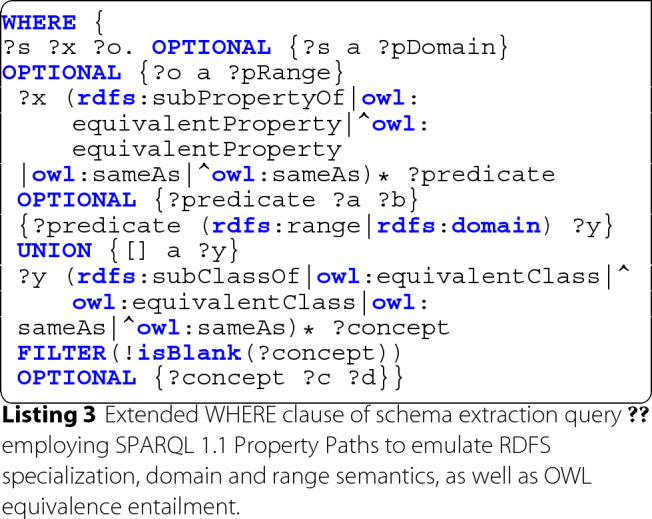


The query constructs a graph, which in addition to all instantiated RDFS classes and RDF properties (and their direct properties) includes generalizations and equivalent resources of those via RDFS and OWL semantics.

For both properties and classes, we resolve corresponding generalizations directly using the relevant RDFS entailment patterns (rdfs5, rdfs7, rdfs9, rdfs11) [53] and concept equivalences using OWL’s owl:equivalentClass, owl:equivalentProperty and owl:sameAs predicates [54] in lines 5 and 9. While owl:sameAs is only supposed to be used for the declaration of equivalence between individuals, it is commonly misused in practice and as such deliberately included in this query.

rdfs:Class annotations are further inferred following RDFS entailment rules rdfs2 and rdfs3 [53] from rdfs:domain and rdfs:range properties declared on instantiated rdf:Property resources (line 7).

**Fig. 3:**
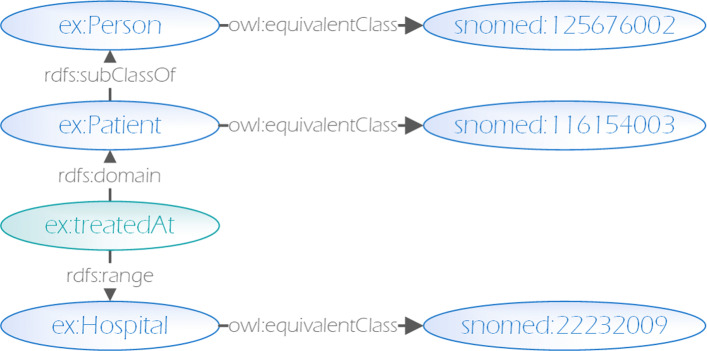
Locally inferred relevant schema extracted from example 1

When applying this extraction approach to the dataset depicted in Fig. 1, we end up with the relevant schema depicted in Fig. 3. As before, classes are highlighted in blue and properties in green.

Following the extracted schema, it is now also possible to query for instances of the hospital and person classes, as well as a number of equivalent SNOMEDCT vocabulary terms.

### Employing terminology services

In practice, individual SPARQL endpoints providing access to individual datasets cannot be (and are not) burdened with serving all vocabularies and terminologies used in the dataset and related to those. That is the purpose of specialized terminology services and vocabulary catalogs, such as the aforementioned LOV and BioPortal projects.

In order to resolve equivalences and generalizations across vocabularies, it is thus possible to make use of the SPARQL 1.1 Federated Query protocol [60, 61] in order to entail additional schema triples using external terminology services. The query depicted in Listing 4 employs federated queries to the SPARQL endpoint http://example.org/terminology in order to accomplish this. The query further explicitly filters out all subject that are blank nodes in order to avoid renaming and resolution issues between blank nodes from different sources (c.f. [60]).

While the approach follows the same principles as the previously introduced local inference (c.f. Listing 3), here each inference step also includes results from the external terminology service. As such, following the example from before, the extracted schema would now also include all inferred knowledge from the SNOMEDCT vocabulary as well as any vocabulary known to the terminology service that declares equivalences with SNOMEDCT.

In some cases, such as with rare diseases, even the limited communication with remote terminology services might affect data privacy, since the instantiation of certain very rare classes or predicates might in itself reveal private data. In such cases a local terminology service can be employed, i.e. by creating a local deployment of the LOV service or by providing local copies of the relevant full vocabularies. Nevertheless, sharing of the extracted schema in such cases may still require additional considerations.

Unfortunately, current implementations of federated SPARQL queries still typically incur large performance penalties by using suboptimal resolution strategies. As such, in practice, it is often helpful to manually decompose the single query into multiple query steps. An exemplary four-step approach using the SPARQL 1.1 UPDATE construct [62, 63] can be found in the supplementary materials^2^, which also includes performance optimized reformulations of the other queries.


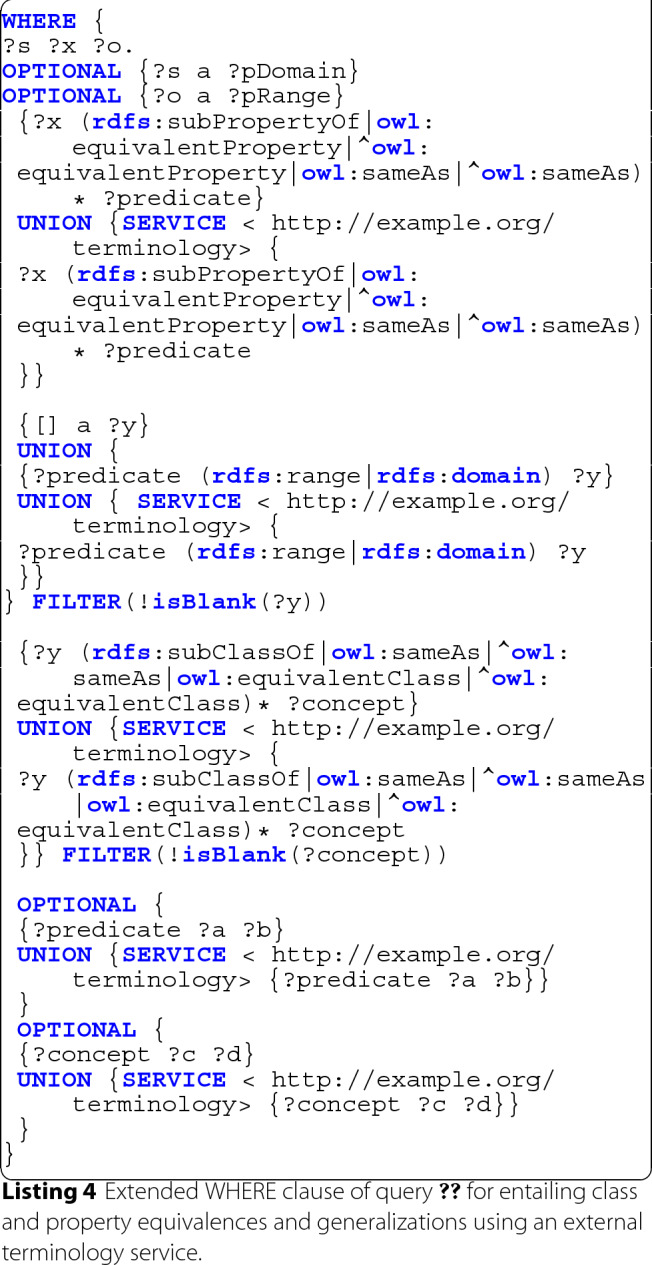


### Schema-aided data selection and integration

Once the schema is extracted, the resulting schema can be publicly or semi-publicly (e.g. with prior authentication) exposed using a dedicated SPARQL endpoint. It is then possible to use existing SPARQL query writing assistance tools (i.e. query builders) such as OWLPath [64], QueryVOWL [58] or VSB [57] together with the extracted schema for schema introspection aided design of data selection and integration queries without direct access to the private dataset. An overview of available tools can be found in [65].

**Fig. 4:**
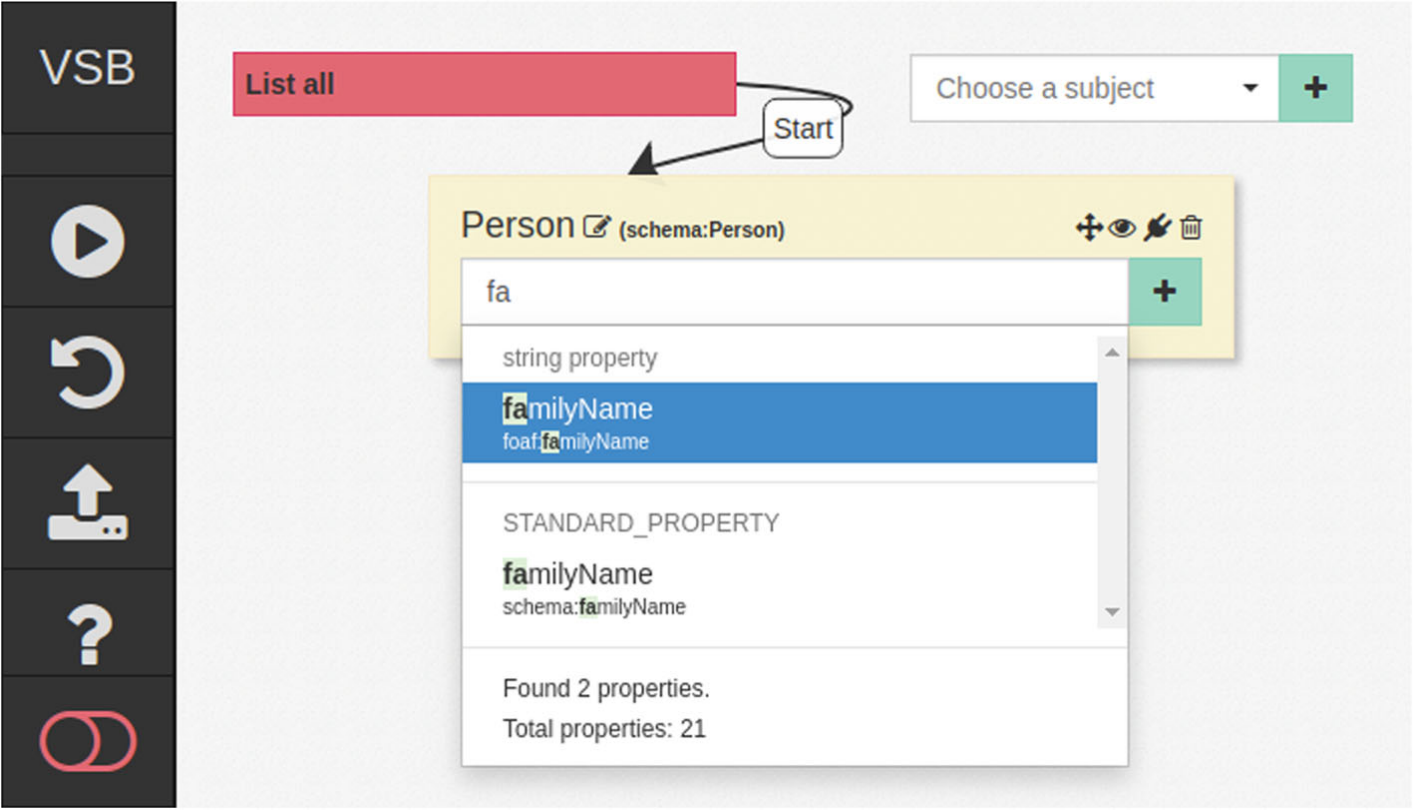
An example of a visual SPARQL query builder tool interacting with the schema introspection endpoint to enable assisted query design

Figure 4 depicts a screenshot of the visual query builder VSB [57], configured to employ introspection of a schema extracted using the “locally inferred” approach, as conducted in the following evaluation. Corresponding instructions for schema extraction and deployment can be found in the supplementary materials. This example illustrates how introspection of the public schema allows for the automated suggestion and autocompletion-assisted search for available properties and classes, as well as the relations between them, enabling easy query writing through interactive schema exploration. In the depicted case, the user is interested in instances of the schema:Person class and provided with a list of property suggestions for the search string “fa”, as available in the original private data.

Such tools may optionally also employ the provided schema in order to construct SPARQL 1.1 queries that can resolve term generalizations and equivalences following the semantics of the extracted schema. As such, the user does not have to rely upon proper entailment support of the dataset SPARQL endpoint but can construct explicit queries that specify the relevant equivalences, further enabling ad-hoc data integration queries through the provided resource equivalences.

As such, e.g. in the example depicted in Fig. 3, it is likely that the private data endpoint does not support entailment. Thus, the query must be constructed in a way to account for the semantic implications of the schema. For example, in order to find all persons, one would have to query not only for all instances of the person class, but also for all instances of its equivalent classes, subclasses, their equivalent classes, as well as those that occur as subject or object of a property with corresponding domain or range, in this case subject of a triple with ex:treatedAt predicate. Query builders and query writing assistance tools can however automatically construct queries accounting for this without burdening the user. Such queries thus allow for the ad-hoc integration of data encoded with different ontologies and standards, based only on the previously extracted schema.

**Fig. 5:**
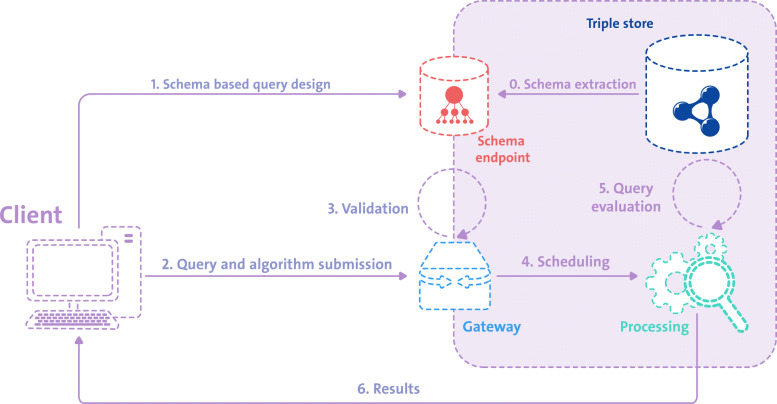
Workflow of the proposed architecture

**System architecture** The workflow of the proposed architecture is illustrated in Fig. 5, which depicts the communication between client and data provider over a public network. In this scenario, the data provider’s internal communication within its private network is highlighted by the bounding box.

In preparation for client usage, the schema of the sensitive data stored in the private triple store is extracted in *step 0* using the approach presented above and deployed to a publicly accessible schema endpoint.

Since the private data store remains inaccessible from outside its private network at all times, the schema extraction has to be conducted by the data provider herself. This could either be done by manually extracting the schema on-demand, e.g. using the four-step “LOV inferred” schema extraction approach employing the SPARQL Update construct, by automatically running a corresponding extraction script in regular time intervals or by creating a “schema view” for the data store, which can then directly be queried by data consumers.

Once the schema endpoint is available, the client can start to create a SPARQL query in *step 1*, using a query builder of their choice in conjunction with the schema endpoint for introspection. The query is then sent to a submission endpoint acting as the gateway between the data provider and the client in *step 2*. For the scope of this work, we assume that this requests includes algorithmic means of data anonymization, ensuring its results are no longer privacy sensitive and that validation is done manually.

Once validated, the request is scheduled in *step 4* for processing within a secure enclave (processing), where the query and algorithm are evaluated (*step 5*). This is analogous to the approach proposed by Jochems et al. [47] and Deist et al. [48] as detailed in the related work section. Finally, only the processing result is returned to the client in step 6 without ever directly granting access to the data.

### Evaluation

In order to evaluate the proposed approach, we extract schema information from a synthetic dataset of patient records (PRs), specifically generated in order to illustrate the intended use case, as well as the three corpora GenDR, Orphanet and NCBI Homologene, as distributed through the third release of the interlinked life science data repository Bio2RDF [66].

The PRs dataset contains personal information of 10,000 individuals such as name, birthday and phone number and is published in conjunction with this paper. The dataset was generated using the open source *generatedata* tool^3^ and converted to a corresponding dataset of 15,0000 RDF triples using the SPARQL Generate extension [67, 68]. Half of the records are encoded using the FoaF vocabulary [69] and half using the Schema.org vocabulary [70].

GenDR [71] is a database of genes associated with dietary restriction (DR), intended to facilitate research on the genetic and molecular mechanisms of DR-induced life-extension.

Orphanet[72] is a database of information on rare diseases and orphan drugs for all publics, intended for the improvement of the diagnosis, care and treatment of patients with rare diseases.

HomoloGene [73] is a database of homolog sequence relationships between 20 completely sequenced and annotated eukaryotic genomes.

GenDR, Orphanet and Homologene respectively provide custom vocabulary definitions describing their data encoding and semantics.

All four datasets were separately deployed to a private triple store and relevant schema extracted using the three presented direct extraction methods, i.e. extracting only directly instantiated properties and classes using query 2, using local inference together with the respectively employed vocabularies (such as FoaF and Schema.org definitions for the PRs dataset) via query 3 and finally using the LOV terminology server via query 4. The employed data and scripts may be found in the supplemental materials.

## Results

In order to evaluate the effectiveness of the schema extraction process, we employ the HCLS core statistical measures [32] to compare the characteristics of the vocabularies, datasets and the extracted schema. Table 1 lists results for the entire Linked Open Vocabularies dataset (employed in the terminology service), the full datasets PRs, GenDR, Orphanet and Homologene, the respective complete vocabularies employed for coding the datasets as well as the three extracted schemata per dataset.

**Table 1:** HCLS core statistics [32] of evaluated datasets, vocabularies and extracted schemata

	**HCLS Metric**	**6.6.1.1**	**6.6.1.2**	**6.6.1.3**	**6.6.1.4**	**6.6.1.5**	**6.6.1.6**	**6.6.1.7**
	number of unique	triples	typed entities	subjects	properties	objects	classes	literals
**LOV**	Vocabulary Corpus	833834	129827	171168	1209	145498	1469	180680
**PRs**	Dataset	150000	20000	20000	20	10003	3	70717
*Vocabulary*	schema.org (1)	8427	1617	1619	15	476	31	3193
	foaf (2)	631	84	86	15	38	9	154
	merge of (1), (2)	9058	1701	1705	23	508	38	3335
*Schema*	directly instantiated	47	23	23	3	5	2	0
	locally inferred	576	95	95	13	71	9	118
	LOV inferred	2345	208	208	87	379	16	850
**GenDR**	Dataset	11609	1123	1123	24	1232	13	5158
*Vocabulary*	GenDR Vocabulary	192	20	20	8	6	5	116
*Schema*	directly instantiated	361	37	37	10	16	7	105
	locally inferred	380	37	37	10	16	7	124
	LOV inferred	911	71	71	58	127	12	370
**Orphanet**	Dataset	377947	28871	28871	38	42891	29	144773
*Vocabulary*	Orphanet Vocabulary	402	40	40	9	7	5	239
*Schema*	directly instantiated	799	67	67	12	41	7	217
	locally inferred	840	68	68	12	41	7	256
	LOV inferred	1380	102	102	59	153	12	506
**Homologene**	Dataset	7189742	869981	869981	14	1420471	10	2865019
*Vocabulary*	Homologene Vocabulary	62	7	7	8	6	5	38
*Schema*	directly instantiated	184	24	24	10	13	7	40
	locally inferred	190	24	24	10	13	7	46
	LOV inferred	721	58	58	58	124	12	292

On first sight, the results clearly show that for all three approaches, the number of extracted triples is significantly lower compared to the respective combined source data, thus reducing the cognitive and computational effort required for schema introspection.

For the directly instantiated properties and classes, we have to compare the extracted schema directly with the full dataset, since there is no other available vocabulary definition in the data source to compare it to. For the PRs dataset, only 47 instead of 15,0000 (i.e. about 0.03% of the number of triples contained in the full dataset) are included in the schema. Nevertheless, manual validation shows that the 23 subjects are exactly the 20 properties and three classes found in the full dataset.

Similarly, with 361, 799 and 184 triples, the size of the extracted schema for GenDR, Orphanet and Homologene datasets is about 3.11%, 0.21% and 0.03% of the respective full dataset. However, compared to the triple counts of the corresponding authoritative vocabularies, there is a significant amount of additional information in the extracted schema with 361:192 (∼88% overhead), 799:402 (∼99% overhead) and 184:62 (∼197% overhead) triples. This characteristic is proportionally reflected in the number of typed entities and subjects extracted with 37:20 (+85%), 67:40 (+67.5%) and 24:7 (+200%). Closer inspection reveals that the additional subjects in the extracted schema are in fact additional properties and classes in the dataset, which are not included in the respective authoritative dataset vocabulary but stem from usage of terms from additional vocabularies within the dataset. As such, reliance onto the data model specified in the authoritative vocabulary when creating data queries, could actually hinder making proper usage of the full available data, while the extracted schema more closely reflects the actual data structure at hand.

Manual validation (c.f. supplementary materials) shows that for the GenDR and Homologene datasets all subjects of the authoritative vocabulary are also included in the extracted schema. For Orphanet all but one are included, the http://bio2rdf.org/orphanet_vocabulary:Disorder-Gene-Association class, which itself does not occur in the dataset but is a superclass of 8 instantiated and correctly included classes. This superclass is however included in the “locally inferred” version of the extracted schema and thus illustrates the proper functioning of the rdfs:subClassOf inference. Since the validation further shows, that for all extracted schemata, the triples contained in the “directly instantiated” schema are a subset of those in the “locally inferred” one, which are in turn a subset of the “LOV inferred” schema, all subjects ocurring in the authoritative vocabulary are also contained in all other extracted schemata.

With 576 triples, the “locally inferred” schema of the PRs dataset is about 6.36% of the size of the union of the full employed vocabularies and a superset of the previously extracted schema of directly instantiated properties and classes. As intended, only the subset of the full vocabularies that actually describes the private dataset (and as such is actually relevant to the data) is extracted, allowing for focused query design based on only the relevant schema, thus saving cognitive as well as computational effort during schema introspection. Manual validation supports the correctness and completeness of the extracted schema.

Similarly, the “locally inferred” schemata of GenDR, Orphanet and Homologene are extended versions of their respective “locally instantiated” variants, enriched by relevant semantically inferred knowledge from the respective full authoratative vocabularies, such as the entailment of generalizations and equivalent classes and properties.

Since these extracted schemata also contain explicit equivalence information (for example between the foaf:Person and schema:Person, which is in this case only declared in the schema.org vocabulary) it is possible to explicitly design queries considering the corresponding implications at query design time without relying upon inference support of the SPARQL endpoints. As such it may provide an additional building block for enabling efficient interoperability across different data codings.

Finally the schema inferred using the central LOV terminology service extends the “locally inferred” schema further by entailing additional schema equivalences, generalizations and knowledge. The extracted schema for the PRs dataset consists of 2345 triples, which roughly equals 0.28 percent of the entire LOV corpus and is yet again a superset of the locally inferred schema. Overall it contains triples occurring in 265 of the 648 vocabularies that make up the entire LOV dataset. As such it provides a valuable source for semantic integration of data across various vocabularies used for coding data. Similarly, with 911, 1380 and 721 triples, the “LOV inferred” schemata for the GenDR, Orphanet and Homologene datasets weigh in at about 0.11%, 0.21% and 0.09% compared to the full LOV corpus.

While no in-depth evaluation of the runtime has been conducted, it might be of interest that the extraction of the “LOV inferred” schema takes about one minute for the largest evaluated dataset (Homologene) and under ten seconds for all other datasets, using two Fuseki^4^ SPARQL server processes serving as triple store and terminology server on a single Intel i7-8700k desktop CPU. Hereby the runtime is largely dominated by the explicit construction of rdfs:domain and rdfs:range properties, not by the SPARQL federation to the terminology service, as illustrated by the fact that the extraction of the “locally instantiated” schema took only between two to four seconds less extraction time in all evaluated datasets.

## Discussion

The presented schema extraction and architecture strive to close a gap between owners and consumers of sensitive data. While related work has already provided us with basic infrastructure in order to allow for the processing of data under the owner’s control, to our best knowledge, all existing approaches relied upon a-priori agreement upon a shared schema and data encoding.

In contrast, the approaches presented in this work are capable of extracting relevant (i.e. instantiated) schema from a given RDF dataset with a configurable amount of inferred information based on RDFS and OWL semantics, which can subsequently be used for SPARQL query design without requiring access to the original data.

As such, ongoing research efforts such as the Personal Health Train initiative could benefit significantly from implementing this or a similar approach in order to enable optional interoperability and ad-hoc data integration and re-use. Especially given the challenges of trans-national standardization efforts of vocabularies and data models, the evolution of such standards over time and the need of individual research to diverge from standards, we believe a system such as the one presented in this work to be essential for the realization of effective data reuse.

As illustrated in Fig. 4, the schema extraced using our approach is suitable for usage with existing schema-introspection-assisted SPARQL query writing tools. As such it does enable the formulation of ad-hoc SPARQL data-integration queries against RDF triple stores without requiring direct access to the private data. The approach is compatible with existing Semantic Web-based technologies and in can be employed in conjunction with the presented system architecture for the subsequent execution of such queries in a safe setting under the data provider’s control, i.e. in the context of the PHT initiative. Thus the presented approach can enable the ad-hoc reuse of private data repositories through schema extraction.

While we believe the current approach to be universally applicable to any data domain (just as the underlying RDF data and RDFS/OWL semantics model), many areas are currently lacking authoritative terminology servers with SPARQL endpoints and support for inference or SPARQL 1.1 features required for any manual inference using our methodology. While the presented approaches work well in conjunction with e.g the Linked Open Vocabularies project, compatibility with e.g. the important Bioportal project is hindered by its SPARQL endpoint, which lacks entailment and SPARQL 1.1 support.

Additionally, many vocabularies introduce custom schema semantics that go beyond RDFS and the subset of OWL that we consider in this work. Examples include schema.org’s domainIncludes and rangeIncludes for properties or Wikidata’s own terms for class and property equivalences and concept generalization. While it is easily possible to extend the presented schema extraction mechanism to account for these additional terms, one may nevertheless wonder about the reasonableness of the redefinition of these basic schema concepts in various vocabularies. Nevertheless, as schema definition languages, vocabularies with their own semantics and related requirements evolve, we believe that a flexible solution such as the one presented in this work will only grow more relevant in practical applications in order to bridge the gap between competing systems and standards.

## Conclusion and outlook

In this paper, we proposed an automated way of schema extraction from Linked Data in RDF format which enables the introspection supported development of SPARQL queries without direct access to the actual data. The approach further allows for the extraction of a configurable amount of semantically inferred schema and the resolution of equivalences across multiple vocabularies and standards. As such, it could provide an important building block in order to enable optional interoperability across competing standards and data encodings. Based on existing Semantic Web Technologies and inspired by recently published work in the context of the Personal Health Train initiative, we further presented a system architecture to realize reuse of data locked in private repositories without having to share the actual data. From the users perspective, our approach enables straight forward query formulation against privacy sensitive data sources and successive evaluation of that request in a secure enclave at the data provider’s end.

With this architecture, we can overcome the reliance of previous approaches on agreeing upon shared schema and encoding a priori in favor of more flexible schema extraction and introspection. While the methodology is designed specifically with the context of the PHT in mind, the approach is likely equally applicable to the broader context of semantic data exploration as well. As such, the presented method promises to provide a key building block in enabling efficient reuse of data across a variety of domains. In conjunction with advanced distributed learning and processing systems, the approach could be used in order to overcome existing data sharing hurdles and unlock hidden value in existing data silos.

## Abbreviations

DR: Dietary restriction
EHR: Electronic health record
GDPR: EU general data protection Rules
IRI: Internationalized resource identifier
KDD: Knowledge discovery in databases
LOV: Linked open vocabulary
OBI: Ontology for biomedical Investigations
OWL: Web ontology language
PHT: Personal health train
RDF: Resource description framework
RDFS: RDF schema
SHACL: Shapes constraint language
ShEx: Shape expression language
SPARQL: SPARQL protocol and RDF query language

## Footnotes

1 Likely the definitions would be http://example.org/and http://purl.bioontology.org/ontology/SNOMEDCT/

2 https://github.com/PersonalHealthTrainGermany/schemaExtraction

3 https://github.com/benkeen/generatedata

4 https://jena.apache.org/documentation/fuseki2/index.html
